# A Qualitative Analysis of Urbanization and Menstrual Health Among Young Women

**DOI:** 10.7759/cureus.56390

**Published:** 2024-03-18

**Authors:** Sithal Dalai, D Shobha Malini, Durga M Satapathy, Sithun Patro

**Affiliations:** 1 Community Medicine, Maharaja Krishna Chandra Gajapati Medical College and Hospital, Berhampur, IND; 2 Community Medicine, Jajati Keshari Medical College & Hospital (JKMCH), Jajpur, IND; 3 Community Medicine, Maharaja Krishna Chandra Gajapati Medical College and Hospital, New Delhi, IND; 4 Epidemiology Research, JHPIEGO, New Delhi, IND

**Keywords:** adolescent gynecology, compliance to iron folic acid tablets, qualitative studies, focus group discussion, government health scheme, menstrual health hygiene, inductive thematic analysis, grounded theory

## Abstract

This qualitative study, grounded in theory, employed inductive coding for analysis, focusing on menstrual health among urban women aged 10-25. The research aims to explore the menstrual health status, practices, and beliefs of participants. The research delves into the impact of recent government initiatives on menstrual health and assesses the role of urbanization in shaping evolving menstrual health practices among young girls. Employing in-depth qualitative methods such as interviews and focus group discussions, the study seeks a comprehensive understanding of participants' experiences and perceptions related to menstrual health. The dynamics of women's menstrual experiences are significantly influenced by urbanization, heightened exposure to social media, evolving lifestyles, and government initiatives like the distribution of menstrual products in schools and the enhancement of water, sanitation, and hygiene (WASH) facilities in government institutions. Positive shifts have been observed, including reduced restrictions on menstruating individuals, enhanced access to affordable hygiene products, and improved disposal facilitated by municipal garbage collection services. However, notable gaps persist in basic knowledge about menstruation, hygienic practices, effective interpersonal communication with schoolteachers or community health care workers, and compliance with government programs promoting weekly iron-folic acid supplementation and biannual Albendazole intake, calling for substantial improvement.

## Introduction

Understanding young women's perspectives on menstruation is vital for sexual health education. The onset of menstruation marks a transformative journey into womanhood for adolescent girls and can be traumatic for female adolescents. Unfortunately, in India, taboos, false beliefs, and limited access to effective menstrual hygiene management (MHM) [1] hinder this transition. According to NFHS-5 (2020-2021), 81.5% of women aged 15-24 now use hygienic protection methods [2], a notable increase from NFHS-4 (47.4%). Despite government initiatives like the Menstrual Hygiene Scheme, challenges persist, including supply shortages. The government aims for universal access to safe water, sanitation, and hygiene (WASH) by 2030. Additional schemes like FREEDAYS (India) and KHUSI (Odisha) [3] provide sanitary napkins to below-poverty line women. NGOs like UNICEF and WaterAid work on education, awareness, and access to MHM products. Efforts include campaigns and media, like the movie PADMAN. Schools are urged to become menstruation-friendly by offering napkins, education, and private facilities. However, sustained change requires a strong commitment, implementing holistic guidelines, promoting sustainable products, ensuring proper disposal infrastructure, and addressing cultural stigmas.

Our research aims to conduct an in-depth investigation into the menstrual experiences of young girls in an urban area. The primary objective is to explore whether there have been any changes in socio-cultural factors and identify new elements influencing the menstrual health status of urban girls. This study is motivated by the need to understand the impact of various menstrual hygiene initiatives launched by different organizations. By delving into the lived experiences of urban girls, we intend to contribute valuable insights that can inform and enhance existing menstrual health programs. This research will shed light on the evolving socio-cultural landscape surrounding menstruation and provide a comprehensive understanding of the factors influencing menstrual well-being among young girls in urban settings.

## Materials and methods

The study was part of a quasi-experimental research project consisting of five phases conducted between 2021 and 2023. The first phase involved formative research as a qualitative study, followed by quantitative data collection from schoolgirls in the second phase. The third phase included a menstrual educational intervention at selected schools, and the study concluded with post-intervention and follow-up evaluations of knowledge and practices related to menstrual hygiene. However, this article presents findings solely from the formative phase due to the extensive nature of the overall study. The final, comprehensive results will be available in a subsequent publication. The original study protocol is accessible on the CTRI website under registration number CTRI/2021/11/037916.

The study included girls aged 10-25 from urban slums in Berhampur, regardless of menarche status, to assess pre-menarche social awareness. Pregnant and lactating mothers with lactational amenorrhea were also part of the study to investigate post-pregnancy perception changes. Participants willing to discuss openly were selected for focus group discussions (FDGs), while those preferring privacy were chosen for in-depth interviews (IDIs). Additional interviews were conducted with subsets of participants identified during focus groups, such as school dropouts, pregnant adolescents, and individuals with health conditions like heart disease. Those with comorbid conditions like PCOS or congenital diseases were also included to understand their impact on menstrual health. Unwilling participants and those who did not consent were excluded from the study.

Establishing a strong researcher-participant relationship based on trust and rapport was crucial for sampling and data collection. The research team tried to cultivate trust and a level of familiarity through prior visits to obtain assent and consent, setting up discussion and interview details accompanied by the local NGO. Girls between 10 and 25 years old who had attained menarche and could express themselves in a group were included in FGDs. Shy girls, those with significant histories (school dropouts, teenage pregnancies, comorbidities), and others (teachers, young mothers, community health workers) were selected for IDIs.

Open-ended question guides for FGDs and IDIs explored key topics [4] like menstrual experiences, socio-cultural influences, and life aspects (Interview guide provided in the appendices). Interviews and discussions were conducted in Odia (vernacular language) and Hindi (for the subset of the Muslim population). Semi-structured interviews were audio recorded and transcribed verbatim, with participants having the option to turn off the recorder. For non-recorded interviews, detailed notes were taken. Transcripts were translated into English for analysis using ATLAS.ti. Thematic analysis was employed following the steps suggested by Braun and Clarke [5]. A codebook and themes were created based on the literature and qualitative guide. Original quotes from participants supported the conclusions.

The qualitative analysis utilized a grounded theory approach with inductive coding, integrating existing research on menstrual health with fresh perspectives on evolving demographics, digital progress, and governmental interventions in urban India. Throughout the study, supplementary sub-themes surfaced, encompassing inputs from educators and governmental strategies. Verbatim responses were organised alongside participant numbers (P1, P2, Pn, etc.) and interview identifiers (FGDn, IDIn).

This project was funded by the “Mamta Health Institute for Mother and Child, New Delhi” under the fellowship scheme for the medical postgraduates in India. Ethical permission was obtained from the Institutional Ethical Committee after the grant approval and prior to study commencement. The original study protocol is registered on the Clinical Trial Registry of India. Consent was obtained from selected schools, parents of girls below 18 years, and adult participants, followed by assents from participating girls below 18 years.

## Results

To assess participants' knowledge and hygiene habits, data were collected through FGDs and IDIs until data saturation was achieved. The study included a total of 53 participants in four FGDs and 26 IDIs. FGD participants were young girls aged 10-25 from urban communities, schools, and slums. IDIs targeted a subset of FGD participants with significant menstrual issues, along with schoolteachers, mothers of menstruating girls, and community health workers. Participants were purposefully selected from schools and slums in urban areas with consent. Transcribed data were analyzed to generate codes, and themes were identified. Common codes were merged to form domains, sub-domains and themes listed under the domains in Table 1. Analysis of both IDI and FGD was done together.

**Table 1:** Thematic framework of the focus group discussions

Theme	Sub-theme	Codes
Menstruation and it's management.	Knowledge on Menstruation	Why it occurs
		Age duration
		Normal period
		Pre-menstrual symptom
	Menstrual experiences	Awareness prior to menarche
		First menstrual experience
		Menarche ritual at home
		Menstrual life
		Menstrual symptoms
	Menstrual Hygiene Practice	Menstrual products
		Menstrual waste management
		Hygiene practice
		Home management of symptoms
		Toilet access
	Psycho-social aspect	Cultural taboos and beliefs
		Inter-personal communication
		Health seeking behavior
		Effect on social life
	Government initiatives	Free product (pad and iron tablet) distribution
		IEC and BCC activities

Knowledge on menstruation

Initial questions about the students' knowledge of menstruation focused on what they already knew. Most often, participants did not respond because they were hesitant. Most of them, however, said that they are unaware after being probed further. The physiology of menstruation was largely unknown to them. 

P3 - “every 28 days if eggs are not fertilized in form of blood, it comes out in form of blood after 28 days it called as periods” - FGD1. 

But most believed this is the process of removing impurities out of our body.

P9 - “Impure blood from body goes away once in every month &pure blood comes in replacement of impure blood” -FGD2.

Further when asked about the age group in which it occurs, most replied that it starts around 12 year and occurs till about 60 years of age. 

P10 - “My mother was saying that because of what people eat now, they get it in 8 or 9 year itself. before it was like 13-14-15 years. Because what they used to eat at that time it was normally fresh, but people are whatever they are eating right now... like all those tablets and all... that's why the children are getting it earlier.” - FGD2

When asked to describe the characteristics of a normal period, almost everyone responded that it is free of cramps, pain, and bleeding for a typical amount of time, such as seven days per month. 

P16 - “yes, if there is no pain and like this...it bleeds clearly for seven days then that is normal period and if there is pain and all that is…” - FGD3. 

P7 - “1st day to 4th day normal bleeding, decreases by 5th day, 3-4 pads used in one day, Color should be red in normal period. If black color, then it is abnormal.” - FGD1. 

However, less than half of them knew what the term "pre-menstrual symptom" meant when asked about it. When questioned further, most of them acknowledged having experienced the symptoms. 

P21 - “mood swing, irritation, pain...like this... commonly symptoms occur before periods” - IDI6. 

P13 - “Pimples, pain, abdomen, leg ache before periods during or after periods” - FGD3. 

Participants were questioned about their awareness of menstruation at the time of their menarche. Most people denied having any knowledge of this, however a few people did know about some short of bleeding from their friend or older sister. 

P26 - “No. my friend had said that something like this is going to happen before but did not know that much will happen, she said that you are not old yet, you will know when it happens. My friend was absent for a few days, then said that there was some marriage at home, health was bad, told like this but did not say anything in detail. Said that your time will come then you will know” - IDI11. 

Menstrual life experiences

Then, to learn more about their issues, the participants were asked to explain their menstrual life experiences. They were first questioned regarding their first period-related experience. Almost everyone was terrified, kept their secrets, and avoided telling anyone. Only when it was specifically mentioned, or they were unable to handle it on their own did they turn to their parents.

P12 - “In the morning my family was going out and saw something like this. So, they said that something has happened, they said that we are going to call and tell us the good news. Then I didn't understand. it was like when I came to know that I had it, then I cried a lot. I didn't know anything then. Then I went to daadi (grandmother) and asked. Told daadi that something like this is happening, then daadi said that don’t touch, stand aside, and call your Ammi. I called Ammi (mother). Ammi gave me some kind of pad. Then I used the pad and then I thought everything is fine. Then Ammi said that it happens to all the girls, it is not a problem...I was 15 years, maybe in the ninth class back then” - FGD2. 

P22 - “When my elder sister had her first, there was a grand celebration. Then Papa (father) called everyone, then something like this happened. (thinking)... at 4:00 or 5:00 in the morning. Used to take bath in the morning, apply turmeric every night. Then used to feed something …and mix something in Banana and feed it so that the period does not smell.... after feeding her and brought her to the place to do the ritual, they kept something like coconut-banana and other, in her hand” - IDI7.

Further investigation into their current menstrual cycle was done using several probes, including questions about cycle length, flow days, any accompanying symptoms, etc. To this, almost everyone said that their period was generally uneventful. Few people experienced problems including severe bleeding, acne, backaches that required rest, etc. 

P20 - “9day. It stops by 9day. Bleeds till day 5 then decreases gradually.” - FGD4. 

P27 - “first few days I get something like white that comes out. then in middle of the month also some like watery discharge” - IDI12. 

Menstrual hygiene practice 

When asked what kind of menstrual products they used, it became apparent that roughly a little shy of half relied only on the free pads provided by their school, while a significant fraction of them still preferred to purchase higher-quality goods from stores, even if they were receiving the free pads. Everyone in the group agreed that the shops would first wrap the napkin pack in a newspaper before putting it in a piece of black polythene and giving it to the customer. Only a small percentage have used alternative products like tampons or menstruation cups. 

P9 - …” ours…Asha Didi brings for us. She will bring it when we ask… then we buy from medicine store in emergency…. we give her money to buy the pads.”. - FGD2. 

P6 - “the shopkeeper wraps in newspaper then puts it in black plastic bag” - FGD1.

P22 - “now using pads, but initially using cloth. Old clothes” - IDI7.

When asked how used napkins were disposed of, the majority replied that they were thrown outside, some in open drains, and only a small minority would wait for the garbage truck to pick them up. Few people had previously utilised the incinerator at the school or the residential complex. 

P11 - “there is a box in school. When the pad is full, then you must put it in the box, then close it and switch the green button on. Then it will burn, and smoke will go out…. I have never used it. Just have seen it being used… normally I come home from school to change… Once I had change at school. There is an open drain behind our school. I threw it there.” -FGD2.

P18 - “in drain…garbage van comes…but we don’t. We throw in the drain…as mommy must go to shop every morning (shop owner). so, she throws it by 6a.m. The van then comes later…8.30am around” - FGD3.

P35 - “bury in the backyard... (). Yes, we have some space in our backyard” - IDI17. 

According to most explanations, everyone bathes every day and only washes their hands after changing a pad when they are menstruating. The frequency of pad changes, nevertheless, wasn't enough. Everyone has access to a bathroom both at home and at school.

P3 - “we don’t wash hand before. Just after... With water. Soap if it's there” - FGD2.

P7 - “I use the warm water for intimate wash... clean the toilet before use” - FGD1. 

When asked about their understanding of how to handle mild to moderate menstrual symptoms at home, the majority knew to use hot water, but they all believed that a lack of physical activity would lessen symptoms. 

P10 - "if grandma is at home, then she gives methi (Fenugreek). If there is too much pain then… () ...eat methi and drink some water… () ...the pain doesn't disappear completely, it just subsides to some extent.” - FGD2. 

P4 - “while at home, I just sleep with the chunni (a type of cloth) tied around my belly. Don’t do any work” - FGD2.

P2 - “you can use hot water bag, some soothing essential oil like that...no restriction for eating, doing work... Just be in happy mood” - FGD1.

Psycho-social aspect

Every participant had some sort of restriction to follow when it came to the subject of cultural myths and taboos, with the most prevalent being the religious constraint, followed by food, social interaction, and touching people or objects. Most of the beliefs were rooted in the notion that menstruation women were impure. 

P11 - “yes, I don’t go to masjid…. when it happened for the first time, mommy said that Chicken should not be eaten, should not eat lentils etc., due to which I am in pain, if it smells, then all those things should not be eaten…on eating those your period will smell bad.” - FGD2.

P6 - “whatever clothes I have used those days, those will be kept separate, after 6 days... take early bath on day 3, wash the 3day cloth then... shouldn’t sleep during daytime... take bath with cow dung and turmeric before sunrise” - FGD2.

P9 - “someone is sitting with legs spread then you should not jump over them…it would worsen the period pain.” - FGD2.

P9 - “by chance if I am wearing any new dress and I got my period then if we give that to the dhobi (washerman), those who take clothes for cleaning, if he cleans it then we can wear them to Temple…no, not even if washed by us. Only washed by dhobi.” - FGD1.

P2 - "sweet induces flow and also coconut water and sour food induces early periods” - FGD1.

P8 - “Not to touch Male persons, priest, and bath with turmeric water on 4th day of menstruation.” - FGD1.

P3 - “we are asked not to go to the garden”-FGD1.

When it comes to discussing menstruation with others, everyone admitted that they avoid doing so due to social shame and shyness. They learn about it from personal experience or social media.

P10 - “I asked mum my mum earlier when I was having too much pain in my stomach that "till when is it going to happen?". my mum said don't say like that. this is good to happen. you are lucky that you are getting it. So, she explained by saying like this and said that talking a lot will make you old. (laughed). So, by the time it is over, I will become old, so I said that it is happening now, I am not able to enjoy it, when I become old then what will I enjoy. (laughed). And she laughed and said that you are just a child. Don’t ask too much”. - FGD2 

P9 - “don't prefer to talk about periods or discuss with other family members” - FGD1.

Only a small number of people had sought aid for health problems associated to menstruation, but they did occasionally receive it. After their periods began, only a few of them were still enrolled at school. Due to peer pressure, some of them chose to skip school for the duration of their bleeding. 

P1 - “I used to have back pain, stomachache…I went to City medical... The doctor gave some medicine. I took them... then felt better… () ...it happened only once.” - FGD2.

P2 - “I don’t go to school those days. If happens at school, then I return home.” - FGD2.

Some of the participants were also found to have dropped out of school during the FGDs. Thus, those subsets of participants were further invited for the IDI to gain in-depth idea about their current situation and the driving force for school dropout.

P14 - “I used to receive during my 6th class. Now we buy our selves … (asked why by the interviewer) ... I don’t go school anymore. (Again asked why) … I just don’t feel good. I am not interested in school anymore.”

Insights from interviews with female schoolteachers and community health workers on menstrual management

IDIs with female schoolteachers and community health workers shed light on their roles in the lives of young girls during menstrual management. The findings revealed that a majority of participants recognized the challenges related to menstruation in adolescent girls. They were well aware of prevalent sociocultural, religious, and hygiene-related menstrual restrictions in their communities. Interestingly, all participants expressed strong support for the pad distribution scheme, considering it highly beneficial.

P37 - “ We receive the pads in bulk at the beginning of a year. We divide that among the students and give it out. For the tablets, as we receive the iron tablets every week, we distribute them every Monday, either at beginning of class or after mid-day meal.”-IDI3 (one of the teachers responsible for pad and drug distribution in school)

P33 - “We can’t force feed the tablets to the students. If anything at all happens, like vomiting or else, the media will come to us for explanation. There will be long administrative struggle, so much paper work etc. No one questions the government who gives the tablet, everyone will come to us. We don’t want extra trouble.” - IDI9 (one of the school headmasters)

P47 - “I just provide the pads at govt price, whoever askes of me or comes to the Anganwadi. No one comes for the iron tablets… the deworming tablets we give from house to house during the programme…not everyone eats them…they say I have diabetes and I am eating other ayurvedic medicine for some other causes…” - IDI15 (one of the Community Health Workers)

Government initiatives

The question of government initiatives and any material or educational assistance they received at school or elsewhere was finally investigated. Most of them consented to receiving pads, iron-folic acid tablets every week, and albendazole for deworming every year. However, just a small number of them were taking the tablets since they were unaware, while only half of them used those pads.

P11 - “first they brought a bottle of tablet and gave each one to eat in school. I ate that time. Then they gave 30-40 tablet to take home and eat. I didn’t… then they gave 2 types of tablets to take home. The single bigger one (albendazole) everyone ate that, threw the smaller ones (DEC tablet).” - FGD2.

P5 - “What was given in school was very thin. it was soaked just in an hour… so I did not use it, gave it to my mother. Then I buy for myself”. - FGD2.

The distribution system of hygiene products and tablets faced a significant impact due to COVID-19, especially amidst the frequent closures of government educational institutions.

P19 - “we used to get it before COVID started. Now we buy ourselves.” - FGD4

The inadequate compliance with the distribution of weekly iron-folic acid tablets and biannual albendazole tablets was attributed to the absence of pre-distribution educational sessions for both students and teachers. These sessions are essential to provide comprehensive information on the necessity of drug consumption, potential side effects, a comparison of risks and benefits, and guidance on accessing local government ccentersin case of side effects. The lack of such educational initiatives contributed to the suboptimal adherence to the tablet distribution program. Moreover, the participants seemed to be unaware of importance of the iron-folic acid tablets, albendazole to their menstrual health.

P18 - “I am afraid to take those. No problem so far. I am just afraid what kind of medication are these and what short of side effect may occur” - FGD1.

Some schools have witnessed school health promotion endeavors led by local NGOs, showcasing a commendable community-driven initiative. However, feedback from group discussions suggests that these sessions often lean towards product-oriented approaches, lacking in-depth discussions on the physiological aspects of health events.

P13 - “The teachers in the school were saying that it is not a matter of hiding it, some people had come from outside also and explained if there is any problem, then tell the family members. There are many such people who came to school” - FGD1.

P10 - “some people came to school with a box, and they took out the thing from the box and they told us how to use. They put so much water on it, it soaked all of it. He said that this one is available in some hospital, in Berhampur and it was also so thin, then he said that you can wear it for 2 days even” - FGD2.

The lack of trust in government medical system was one of the reasons cited by a young mother from the slums for the lower compliance to government initiatives.

P48 - “They just give that paracetamol tablet for whatever problem in the govt hospital (Urban Primary Health Centre- UPHC near the slum). So we don’t go there anymore. We just buy from the medicine store. Last time I went there (UPHC) for my irregular menstruation, they gave me the tablet for not getting pregnant (MALA-N). I was not even married that time. I didn’t know that. I showed the tablets to my neighbours. They told me not to eat it. Those people from govt. medical give anything. I was so ashamed in front of them.” - IDI52

## Discussion

The initial assessment of their knowledge revealed a lack of understanding of the physiology of menstruation, with a prevalent belief that it is a process for removing impurities from the body, just like previous studies all across the world [6,7]. While participants generally identified the onset of menstruation around the age of 12, awareness of pre-menstrual symptoms was limited. Despite experiencing premenstrual symptoms, they struggled to differentiate between normal physiological (mild pain, mid-cycle watery discharge, mood change) and pathological symptoms (severe pain, heavy bleeding, foul-smelling discharge, itching, malaise due to decreased iron reserve), which were not much discussed in previous studies.

In terms of menstrual hygiene practices, a significant number of people relied on freely provided pads distributed by the government in schools and at low cost through Anganwadi centers, facilitated by community health workers. The observed disposal methods were diverse, while the utilization of alternative products remained limited. This stands in contrast to studies in different parts of India, where challenges such as insufficient access to menstrual products and inadequate disposal facilities were reported [8]. Disposal habits revealed that numerous participants disposed of used sanitary napkins, encompassing both disposable and reusable pads, outdoors. Some chose to discard them in open drains, while a minor percentage awaited collection by garbage trucks. Notably, prior studies have often mentioned traditional disposal methods like open spaces and burial [9], but there is a lack of discussion on enhanced area-specific disposal practices in the existing literature.

Period poverty stands as a crucial yet often overlooked concern in the realm of reproductive health and rights for women of reproductive age [10]. The influence of COVID-19 has exacerbated challenges, particularly in the supply chain of menstrual products within schools, leading to a notable rise in out-of-pocket expenses for menstrual hygiene management (MHM). Additionally, some individuals have resorted to using traditional cloth methods for menstrual management, as indicated in a prior study [11].

Privacy did not emerge as a significant concern for participants in both slums and schools, thanks to the government-provided toilets, as opposed to previous studies [12,13]. However, despite the well-maintained infrastructure of these built toilets, there remains ample room for improvement in ensuring hygienic conditions at these locations.

The psycho-social aspect revealed cultural myths and taboos, with restrictions on activities such as eating, social interactions, touching people or objects, and adherence to specific rules, etc., like other studies [13,14], rooted in the perception of menstruating women as impure. Participants expressed social shame and shyness when discussing menstruation with others, relying on personal experience or social media for information. The investigation into menstrual experiences revealed the initial fear and secrecy surrounding participants' first periods, sometimes prompting them to seek guidance from their parents. In a few cases, this secrecy and discomfort led to school dropouts. The reasons for these dropouts varied; while some were attributed to the uneasiness associated with menarche, others were simply due to a lack of motivation to pursue further education. As opposed to literature [10,14], bullying was not a concern for this study participants. Rather their own fear of staining and physical discomfort during menstruation contributed more to the school absenteeism.

Despite the integration of menstrual health promotion into the school curriculum, a noteworthy observation was made teachers and the community health workers often lacked self-efficacy in addressing the challenges and health concerns related to MHM during discussions with their students and beneficiaries in the community [15].

While the government's efforts to provide menstrual health support have been recognized, several challenges contribute to suboptimal compliance. The lack of pre-distribution educational sessions, coupled with issues such as subpar product quality, interrupted supply chains, and limited accessibility to affordable, higher-quality menstrual products from commercial centers, have been identified as key factors. These challenges, as indicated by a previous study in India, underline the need for a more comprehensive approach to ensure the success and effectiveness of government initiatives in addressing menstrual health issues [15]. While local NGOs demonstrated commendable efforts in school health promotion, the study identified a need for more comprehensive discussions on the physiological aspects and practical aspects of menstruation in such sessions.

The study's outcomes may not apply universally to all urban young menstruating girls as the research was conducted in a limited urban setting with minimal sociocultural diversity. Furthermore, due to COVID-19 constraints, certain minor community viewpoints were unexplored. Additionally, the study lacked the input of district health officials and field staff regarding their roles and regulations in menstrual health management, likely due to time constraint because of their increased workload during the pandemic.

## Conclusions

The dynamics of women's menstrual experiences are significantly influenced by urbanization, increased exposure to social media, evolving lifestyles, and government initiatives such as the distribution of menstrual products in schools and the enhancement of WASH facilities in government institutions. While there have been positive shifts, including reduced restrictions on menstruating individuals, improved access to affordable hygiene products, and proper disposal facilitated by municipal garbage collection services, there remain notable gaps. Basic knowledge about menstruation, hygienic practices, effective interpersonal communication with schoolteachers or community health care workers, and compliance with government programs promoting weekly iron-folic acid supplementation and biannual Albendazole intake still require substantial improvement.

## Appendices

**Table 2:** FGD guide on menstrual hygiene

QUESTIONS
Let’s discuss about the menstrual life. (Like at which age it started, where, what happened that day, whom did you approach first, what was your reaction to the first episode?) Summery:
About puberty ritual at home. (Any specific function for the coming-of-age ceremony? If yes then ask for details)? Summery:
Physiology: What do you think is menstruation? Why does this occur? Summery:
Relation to age: Do you know at what age it should start? Also, till which age it occurs? Then when it does not occur? Summery:
Normal period: What do you think is a normal period? Like the color of blood, how many times do you change your pad, how many days do you bleed, any pain or other discomfort? Summery:
Sanitary absorbent used: How are you procuring your sanitary products? What type are you using? How do you dispose of your used pads? Anyone using cloths? If yes then how do you take care of it afterward? Have you ever experimented with any new interesting products? How much do you spend on an avg monthly for this? Are you able to afford it? Any problem with this matter? Summery:
Hygiene measures: How is the toilet facility at home/school or other places you go to during that time? Do you face any problem? Summery:
Seeking help: When should you go for medical consultation? Do you have any idea? Summery:
Menstrual health issues: Have you ever heard about Pre-menstrual symptom? What do you think this is? Summery:
Home managment of pain: Do you know how to manage menstrual pain / discomfort at home? Summery:
Social aspect: What Issues do you face with stigmas associated with menstruation? Summery:
